# Comparative Proteomic Analysis of Aluminum Tolerance in Tibetan Wild and Cultivated Barleys

**DOI:** 10.1371/journal.pone.0063428

**Published:** 2013-05-14

**Authors:** Huaxin Dai, Fangbin Cao, Xianhong Chen, Mian Zhang, Imrul Mosaddek Ahmed, Zhong-Hua Chen, Chengdao Li, Guoping Zhang, Feibo Wu

**Affiliations:** 1 Department of Agronomy, College of Agriculture and Biotechnology, Zijingang Campus, Zhejiang University, Hangzhou, P.R. China; 2 School of Science and Health, Hawkesbury Campus, University of Western Sydney, New South Wales, Australia; 3 Department of Agriculture, Government of Western Australia, South Perth, Western Australia, Australia; Nanjing Agricultural University, China

## Abstract

Aluminum (Al) toxicity is a major limiting factor for plant production in acid soils. Wild barley germplasm is rich in genetic diversity and may provide elite genes for crop Al tolerance improvement. The hydroponic-experiments were performed to compare proteomic and transcriptional characteristics of two contrasting Tibetan wild barley genotypes Al- resistant/tolerant XZ16 and Al-sensitive XZ61 as well as Al-resistant *cv*. Dayton. Results showed that XZ16 had less Al uptake and translocation than XZ61 and Dayton under Al stress. Thirty-five Al-tolerance/resistance-associated proteins were identified and categorized mainly in metabolism, energy, cell growth/division, protein biosynthesis, protein destination/storage, transporter, signal transduction, disease/defense, etc. Among them, 30 were mapped on barley genome, with 16 proteins being exclusively up-regulated by Al stress in XZ16, including 4 proteins (S-adenosylmethionine-synthase 3, ATP synthase beta subunit, triosephosphate isomerase, Bp2A) specifically expressed in XZ16 but not Dayton. The findings highlighted the significance of specific-proteins associated with Al tolerance, and verified Tibetan wild barley as a novel genetic resource for Al tolerance.

## Introduction

Ionic aluminum (Al^3+^), highly toxic to plant growth, is a major factor limiting crop productivity on acid soils [1]. The strategies for maintaining production on acid soils include lime application to raise soil pH and use of plants with high tolerance to acid soils. Development and planting of Al tolerant cultivars is a cost-effective and practically acceptable approach for full utilization of acid soil [2].

Barley (*Hordeum vulgare* L.) is one of the most Al-sensitive species among small grain cereals [3]. Al toxicity limits the growth and productivity of barley on acid soils and its expansion as a crop into many agricultural areas in the world [4]. In order to breed barley cultivars tolerant to Al toxicity, it is especially important to identify genetic resources with Al tolerance. Wild barley germplasm is rich in useful genes for crop improvement [5]. Tibetan annual wild barley from Qinghai-Tibet Plateau is regarded as one of the progenitors of cultivated barley and is rich in genetic diversity [6]. We successfully identified Tibetan wild annual barley genotypes with high tolerance to both low pH and Al stress [7]. However, their underlying physiological and molecular mechanisms in Al tolerance remain unclear.

Comparative proteomic analysis and bioinformatics techniques provide powerful tools to identify proteins expressed under abiotic stress [8]. Root proteomic analysis showed that proteins involved in stress defense, metabolisms and signal transduction were important for soybean [9], tomato [10] and Arabidopsis [11] plants survival under Al toxicity. However, only limited information is available on Al accumulation/translocation and Al tolerance mechanisms in barley. Moreover, physiological and proteomic responses to Al stress in Tibetan wild barley genotypes have never been investigated and compared with elite Al-tolerant barley cultivars. Thus, precise knowledge of the proteomic basis is required to dissect the mechanisms underlying acid/Al tolerance in wild barley. In the present study we examined stress-specific proteins for acid/Al tolerance in wild barley by comparing the proteomic responses of the two Tibetan wild barley genotypes XZ16 (high acid/Al tolerant), XZ61 (acid/Al sensitive) and Al-tolerant *cv.* Dayton using two-dimensional gel electrophoresis (2-D) and mass spectrometry (MS). These results are useful to better understand the mechanisms of Al tolerance in barley, and provide an effective pathway for the exploration of Al-tolerant genes in plants.

## Materials and Methods

### Plant Materials and Experimental Design

Hydroponic experiments were performed using two Tibetan annual wild barley XZ16 and XZ61 (*H. vulgare* L. *ssp. spontaneum*), acid/Al- tolerant and sensitive genotypes, respectively, and one Al-tolerant-cultivar Dayton. Seeds were surface sterilized in 1% H_2_O_2_ for 30 min, rinsed with distilled water, and then germinated in sterilized moist quartz sand in an incubator at 20±1°C. Seven-day-old uniform seedlings were transplanted into 5-L containers filled with 4.5 L basal nutrient solution (BNS). The composition of BNS was described in Wu *et al*. [12]. The container was covered with a polystyrol plate with 7 evenly spaced holes (2 plants per hole) and placed in a greenhouse. Solution was continuously aerated with pumps and renewed daily after Al addition. The solution pH was adjusted to 5.8±0.1 with NaOH or HCl, as required.

On the day 7 after transplantation, seedlings were cultured for 1 d in 0.5 mM CaCl_2_ at pH 4.3, and then exposed to 0, 50 or 200 µM Al in 0.5 mM CaCl_2_ at pH 4.3 for 24 h. The plantlets were kept in a growth room at 25/20°C with a 14/10 h (day/night) photoperiod and irradiance of 340 µmols m^–2^ s^–1^ light intensity. A split-plot design was adopted with treatment as the main plot and genotype as sub-plot with five replicates in each treatment. At 3 days after treatment, plants were harvested from each treatment, and roots were washed with distilled water thoroughly and collected for two 2-DE experiments and qRT-PCR analysis. Dry weights of the plants were determined and used for Al concentration.

### Determination of Al Concentration and Root Al Distribution

Al concentrations was determined after digestion in an acid mixture (HNO_3_:HClO_4_ = 4∶1, v/v) at 150°C for 10 h, using inductively coupled plasma atomic emission spectrometry (ICP/AES) (Thermo Jarrel Ash, San Jose, CA).

Morin staining for Al in the root tip region was determined according to Zheng et al. [13]. Briefly, 1-cm root tips were stained with 100 mM morin (Sigma-Aldrich, St. Louis, MO, USA) in 10 mM MES buffer (pH 5.5) for 30 min. Images were acquired using a Leica TCS SP2 confocal laser scanning microscope (Leica Microsystems, Heidelberg, Germany) with excitation at 420 nm and emission at 515 nm.

### Protein Extraction, Quantification, Visualization and Image Analysis

Total root protein extracts were prepared essentially according to phenol extraction method [14] with minor modification. Root sample (3 g) of control and Al treated plants were grounded in a mortar separately to a fine powder in liquid nitrogen and homogenized in an extraction buffer containing 30 mg PVPP. The homogenate was suspended in 7 ml ice-cold phenol extraction buffer (0.7 M sucrose; 0.1 M KCl; 50 mM EDTA,0.5 M Tris-HCl, 1% w/v DTT, pH 7.5; complete protease inhibitor cocktail (Roche Applied Science)) and immediately added 7 ml ice-cold Tris buffered phenol and vortexed for 15 s. The sample was then vortexed for 10 s every 5 min and repeated for six times at 4°C. After centrifugation (30 min, 5000×g, 4°C) the phenolic phase was collected, and the sample was re-extracted with 14 ml of extraction buffer (added with the same volume of phenol extraction as collected items), and vortexed for 10 s every 5 min and repeated for six times at 4°C. After centrifugation (30 min, 5000×g, 4C) the phenolic phase was collected and precipitated overnight with five volumes 100 mM ammonium acetate in methanol at −20°C. After centrifugation at 5000×g for 30 min at 4°C, the supernatant was removed and the pellet was rinsed twice in ice-cold acetone/0.2% DTT. Between the two rinsing steps, the sample was incubated for 60 min at −20°C. The pellet was air-dried, resuspended in 200 µl lysis buffer (7 M urea, 2 M thiourea, 4% CHAPS, 20 mM Tris-HCl, pH7.4, containing 1% w/v DTT; Amersham Biosciences), and vortexed for 1 h at room temperature. Protein concentration was determined by standard Bradford assay using bovine serum albumin as standard (Bio-Rad, Hercules, CA, USA). All chemicals used were, if not further specified in the text, p.a. or electrophoresis grade. All electrophoresis units employed were from Amersham Biosciences.

Protein visualization, image analysis and quantification were determined according to Bah *et al*. [15]. For each sample, at least three independent protein extracts were prepared after each treatment and at least three 2-DE analyses were performed for each protein extract. To analyze the expressed protein patterns, stained gels were scanned and calibrated using a PowerLook1100 scanner (UMAX), followed by analysis of protein spots using GE HealthCare Software (Amersham Biosciences). Spot detection was realized without spot editing. The protein spots were quantified using the % volume criterion. Only those with significant and reproducible changes (*P*<0.05) were considered to be differentially accumulated proteins. The target protein spots were automatically excised from the stained gels and digested with trypsin using a Spot Handling Workstation (Amersham Biosciences). Peptides gel pieces were placed into the EP tube and washed with 1∶1 mixture of 50 µL of 30 mM K_3_Fe(CN)_6_ and 100 mM NaS_2_O_3_ for 10–15 min until completely discolored then washed with 200 µL bi-distilled water (two times for 5 min each). The washed solution was drained and washed with 50% ACN (acetonitrile, Fisher A/0626/17) and 100% ACN rotationally, and then incubated in 25 mM NH_4_HCO_3_ (ammonium bicarbonate, Sigma A6141) for 5 min at 37°C. After leaching out of the incubation solvent, 50% ACN and 100% ACN was rotationally added and dried at 40°C for 5 min respectively. Trypsin digestion was carried out as follows: sequencing-grade porcine trypsin (Promega, Madison, WI, USA) was suspended in 25 mM NH_4_HCO_3_ at a concentration of 12.5 ng per µl to rehydrate the dried gel pieces. The trypsin digestion was carried out for 16 h at 37°C. Peptides were extracted from the digest as follows for three times: 10 µL of 50% ACN containing 0.1% TFA (trifluoroacetic acid, GE HealthCare) was added to each tube and incubated for 5 min at 37°C and the supernatants were transferred to new EP tube. The extracts were pooled and then vacuum concentrated for about 2 h. A solution of peptides was filtrated via Millipore (Millipore ZTC18M096) and mixed with the same volume of a matrix solution consisting of saturated α-cyano-4-hydroxycinnamic acid (CHCA) in 50% ACN containing 0.1% TFA. After the peptides were co-crystallized with CHCA by evaporating organic solvents, tryptic-digested peptide masses were measured using a MALDI-TOF-TOF mass spectrometer (ABI4700 System, USA). All mass spectra were recorded in positive reflector mode and generated by accumulating data from 1000 laser shots. The following threshold criteria and settings were used: detected mass range of 700–3200 Da (optimal resolution for the quality of 1500 Da), using a standard peptide mixture (des-Argl-Bradykinin Mr904.468, Angiotensin I Mr1296.685, Glul-Fihrinopeptide B Mr1570.677, ACTH (1–17) Mr2093.087, ACTH (18–39) Mr2465.199; ACTH (7–38) Mr3657.929) as an external standard calibration, with laser frequency of 50 Hz, repetition rate of 200 HZ, UV wavelength of 355 nm, and accelerated voltage of 20,000 V. Peptide mass fingerprint data were matched to the NCBInr database using Profound program under 50 ppm mass tolerance.

### Peptide and Protein Identification by Database Search

Data were processed using the Data Explorer software (Applied Biosystems) and proteins were unambiguously identified by searching against a comprehensive non-redundant sequence database (NCBInr) using the MASCOT software search engine (http://www.matrixscience.com/cgi/search form.pl?FORMVER = 2&SEARCH = MIS). Folds of increase and decrease in Al exposed *vs* unexposed roots were calculated as treated/control and -control/treated for up- and down-regulated proteins, respectively. For single-peptide identified proteins, up- and down-regulation were assigned when the regulation factors were above 1.5 (p<0.05).

### qRT-PCR Analysis

Total RNA was isolated from roots with the TRIzol reagent following manufacturers’ recommendation (Invitrogen, Karlsruhe, Germany). cDNA samples were assayed by quantitative real time PCR (qRT-PCR) in the iCycler iQTM Real-time PCR Detection System (Bio-Rad, Hercules, CA, USA) using the SYBR Green PCR Master Mix (Applied Biosystems). The PCR conditions consisted of denaturation at 95°C for 3 min, followed by 40 cycles of denaturation at 95°C for 30 s, annealing at 58°C for 45 s and extension at 72°C for 45 s. Gene-specific primers (Table S1) were designed using the Primer Express software (Applied Biosystems). Barley *actin* gene was used as control (AY145451) fw-5′-GACTCTGGTGATGGTGTCAGC-3′, rv-5′-GGCTGGAAGAGGACCTCA-3′.

### Statistical Analysis

Statistical analysis were performed using the Data Processing System (DPS) Software Package [16]. Statistical significance of the data was evaluated by two-way ANOVA using Duncan’s multiple range test (SSR).

## Results

### Tibetan Wild Barley XZ16 Is Highly Tolerant to Al Toxicity

Time of appearance and severity of Al toxicity symptoms differed greatly among the three genotypes (Figure S1). XZ16 was less affected by 24 h exposure to 50 or 200 µM Al (pH 4.3), whereas XZ61 was obviously affected, as reflected by severe root growth inhibition. No significant difference between control and 50 or 200 µM Al stressed plants was found in root DW (dry weight) of XZ16, and the whole plant DW of XZ16 and Dayton. However, root and the whole plant DW of XZ61 decreased by 10.7% and 7.8% (50 µM Al *vs* control) and by 19.1% and 13.5% (200 µM Al *vs* control), respectively.

**Figure 1:**
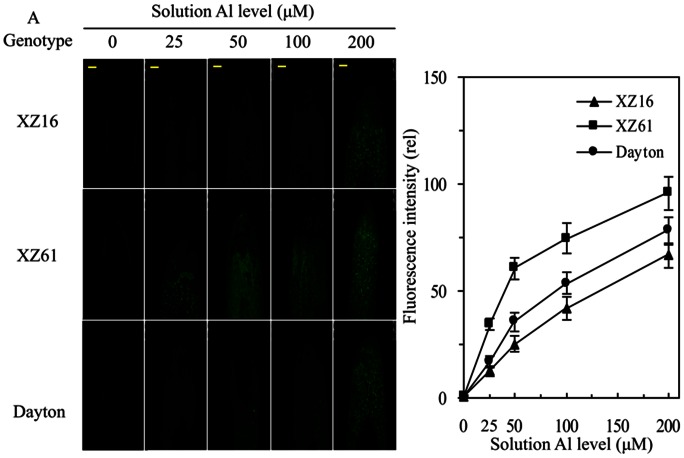
Al localization in barley roots exposed to different Al levels for 24 h. (A) Al was monitored by morin fluorescence using confocal laser scanning microscopy. Fluorescence intensity of image analysis was calculated using Image J software. (B) Seedlings were subjected to 0, 25, 50, 100 and 200 µM AlCl_3_, respectively, in 0.5 mM CaCl_2_ solution at pH 4.3 for 24 h, and then root tips (0–10 mm) were stained with morin. Images shown in the Figures are representative of more than fifteen seedlings per treatment. Bar = 100 µm. Data are means ± SD (n = 5).

**Figure 2:**
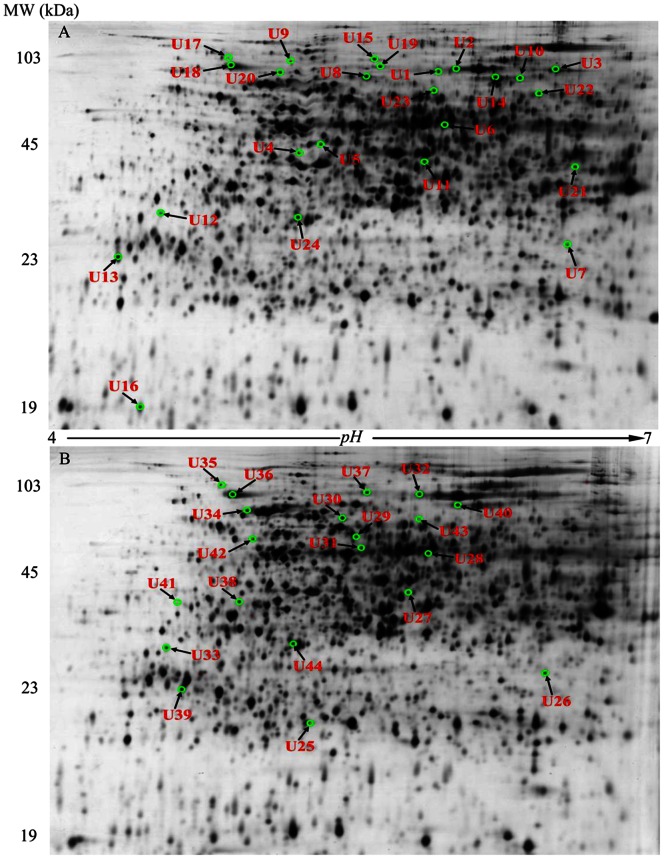
Representative 2-DE maps of root proteins in XZ16 exposed to Al for 24 h. The proteins were isolated from the root of XZ16 exposed to 50 µM (A, upper) and 200 µM (B, below) Al for 24 h. Total proteins were extracted and separated by 2-DE. In IEF, 100 µg proteins were loaded onto pH 4–7 IPG strips (24 cm, linear). SDS-PAGE was performed with 12.5% gels. The spots were visualized by silver staining. Differentially accumulated protein spots are indicated by green sashes. Arrows indicate the differentially expressed protein spots whose expressions were significantly induced (fold changes ≥1.5) or unchanged (−1.50< folds <1.5) in XZ16 but down-regulated (folds <−1.50) in XZ61; or up-regulated in XZ16 but unchanged in XZ61, under 50 µM Al (U1–U24) and 200 µM Al (U25–U44) stress.

Al localization in barley roots exposed to different Al levels for 24 h was monitored by morin fluorescence using confocal laser scanning microscopy (Figure 1A), and fluorescence intensity of image analysis was calculated using Image J software (Figure 2B). Root Al fluorescence showed that root Al concentration increased with increasing external Al levels. XZ16 exhibited similar fluorescent signal in root tips with Dayton, being significantly (p<0.05) less than that of XZ61 in both Al levels.

As to root Al accumulation, there was no significant difference between XZ16 and Dayton, but both exhibited significantly (p<0.05) less accumulation than that of XZ61 in the two Al levels (Figure S2A). Shoot Al accumulation, however, was significantly lower (p<0.05) in XZ16 than in Dayton and XZ61 in response to Al treatments (Figure S2B). The transferring rates from roots to shoots among them were not differed significantly except that Dayton showed 18.2% and 13.6% higher than XZ16 and XZ61 when exposed to 50 and 200 µM Al stress, respectively (Figure S2C). The above results indicate that Tibetan wild barley XZ16 is highly tolerant to Al stress.

### Differential Al-induced Protein Expression in Roots of the Three Genotypes

**Figure 3:**
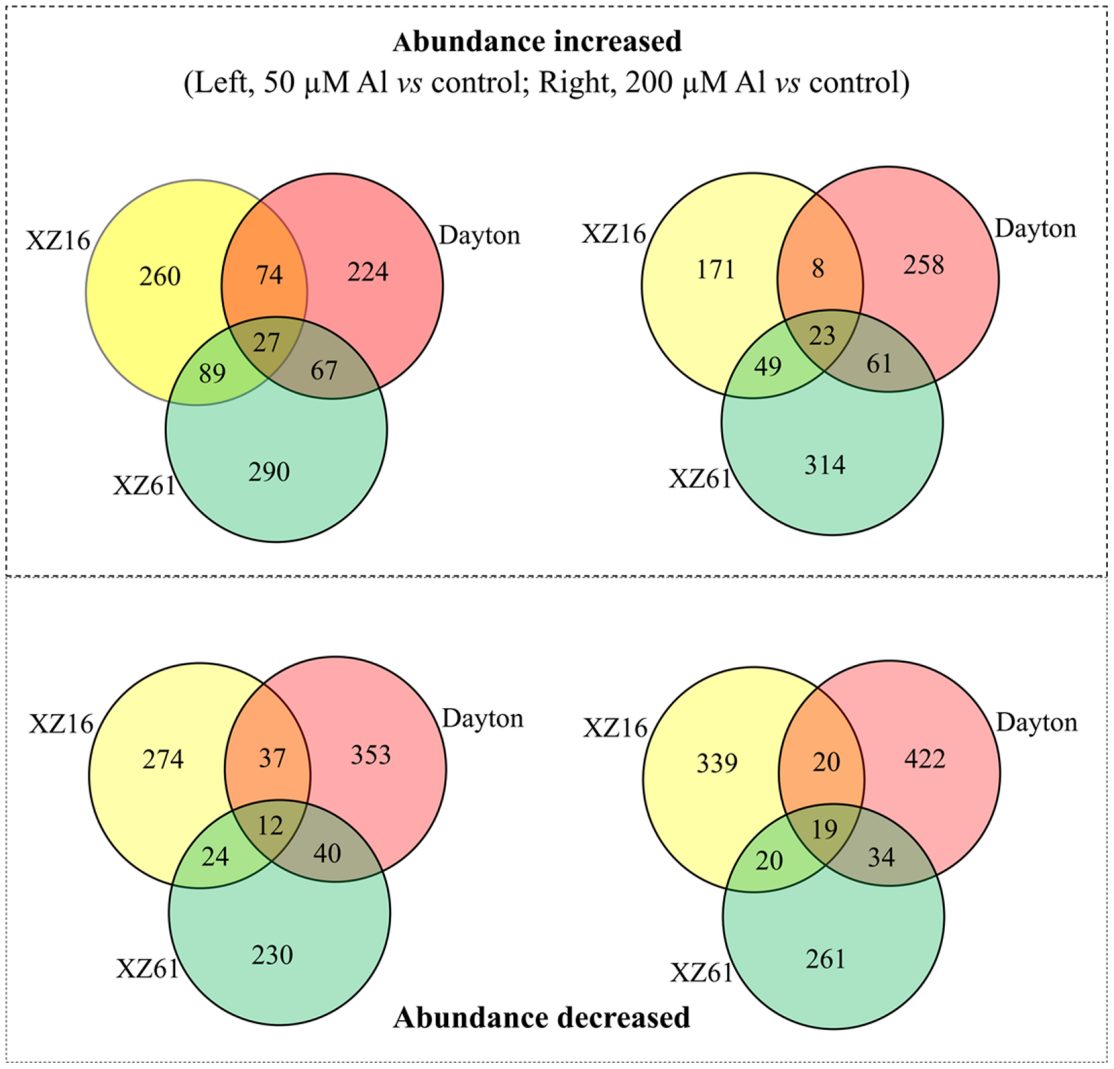
Venn diagram illustrating the expression patterns of Al stress-responsive proteins in roots of XZ16, XZ61 and Dayton. The numbers of differentially expressed spots up- or down-regulated are shown in the different segments. As to protein spots altered by 50 and 200 µM Al stress, 450, 473, 392 spots (50 µM Al vs control) and 251, 447, 350 spots (200 µM Al vs control) were up-regulated in XZ16, XZ61, Dayton, respectively; while 347, 306, 442, and 398, 334, 495 spots down-regulated. The abundance of 101, 31 spots increased in both of XZ16 and Dayton under 50, 200 µM Al stress, respectively, and that of 49, 39 decreased.

Approximately 2268 spots, ranging from 2038 to 2642, were resolved in each of two reproducible SDS-polyacrylamide gels (Figure 2). Protein spots altered by Al stress in Al-tolerant genotypes XZ16 and Dayton and Al-sensitive XZ61 or differentially accumulated among the three genotypes under Al stress were further analyzed and shown in Figure 3. For the protein spots altered by 50 and 200 µM Al stress, 398, 334, 495 spots (50 µM Al *vs* control) and 251, 447, 350 spots (200 µM Al *vs* control) were up-regulated in XZ16, XZ61, Dayton, respectively; while 347, 306, 442, and 398, 334, 495 spots were down-regulated. In XZ16 and Dayton, the abundance of 101 and 31 spots increased and that of 49 and 39 decreased in response to 50 and 200 µM Al, respectively,.

**Figure 4:**
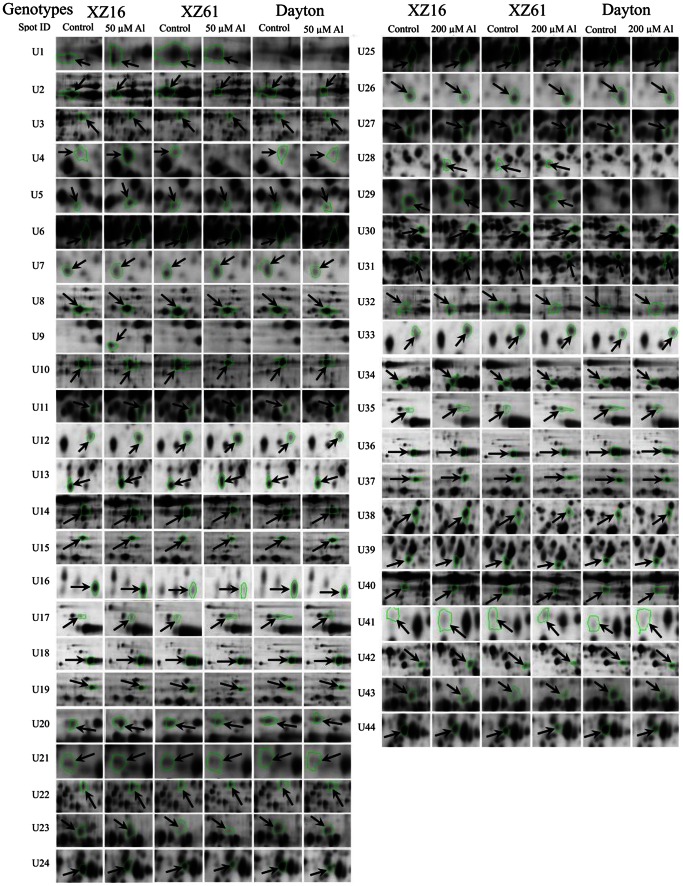
‘Spot view’ of the abundance of differentially expressed proteins in roots of barley seedlings under 50 or 200 µM Al for 24 h. The images of three genotypes: XZ16, XZ61 and Dayton (indicated with green circles) from control and 50 or 200 µM Al (pH 4.3) treated plants. Protein spot ID refers to numbers in Figure 4 and Tables 1, 2.

Al-responsive protein spots (44 spots, U1–U24, U25–U44; Figure 4) were analyzed by MALDI-TOF/TOF MS, and identified by MS/MS data with significant probability (p<0.05). Twenty-four and 20 spots were up-regulated (fold change >1.50) in XZ16 but unaltered/down-regulated in XZ61, or unaltered in XZ16 but down-regulated in XZ61 under 50 (U1–U24, Figure 2A, Table 1) and 200 µM Al (U25–U44, Figure 2B, Table 2), respectively. The resulting spectra of the 44 protein spots (nine spots overlapped at 50 and 200 µM Al, as shown in brackets in Table 2, were identified using MASCOT software search engine against *H. vulgare* and homologous proteins of other green plants in the NCBI non-redundant (nr) protein database and barley ESTs databases (Tables 1 and 2). These proteins were classified into nine groups based on their biochemical functions [17]. The majority of the protein profile was energy (spots U8–U11, U27–U32) and metabolism (U1–U7), followed by protein destination/storage (U14–U19 and U34–U37) and unknown (U21–U24, U41 and U44). The other six minor groups included signal transduction (U20 and U39), cell growth/division related proteins (U12), protein biosynthesis (U13), transporter (U38) and disease/defense (U40). Further comparison of the 44 identified spots with that of Dayton revealed that 16 (Spots U1, U4, U5, U6 (U25), U9, U11 (U27), U12 (U33), U13, U17 (U365), U20, U21, U22, U23 (U43), U28, U29 and U41; Tables 1 and 2) proteins up-regulated in XZ16 were surprisingly down-regulated or unaltered in both Dayton and XZ61. There were a protein uniquely expressed (U9, ATP synthase beta subunit) in XZ16 and three proteins not expressed in Dayton [U1, S-adenosylmethionine synthase 3 (SAMS); U28, a homologue of triosephosphate isomerase (TPI); U29, Bp2A protein]. On the contrary, three proteins (U18, U31, and U40) were slightly up-regulated in Dayton under Al stress, but unaltered in XZ16.

**Table 1:** Proteins whose expression were significantly induced (+) in XZ16 roots but down-regulated (−)/unchanged in XZ61, or unchanged in XZ16 but down-regulated in XZ61 under 50 µM Al stress.

Spot ID	Protein name	Accession number	MW Da	pI	AASC %	MP	Folds (Al *vs* control)		
							XZ16	XZ61	Dayton
	Metabolism								
U1	S-adenosylmethionine synthase 3 [*Hordeum. vulgare* subsp. *vulgare*]	gi|122220777	43138	5.51	42	11	+2.39	−2.44	0.00
U2	Methionine synthase [*H. vulgare* subsp. *vulgare*]	gi|50897038	84794	5.68	35	19	−1.34	−3.39	−2.23
U3	Methionine synthase [*H. vulgare* subsp. *vulgare*]	gi|50897038	84794	5.68	22	12	−1.24	−2.68	+1.02
U4	Glutamine synthetase [*Arabidopsis thaliana*]	gi|228456	47123	6.73	15	7	+6.50	−10^6^	−1.09
U5	γ-glutamylcysteine synthetase [*Triticum aestivum*]	gi|57903694	43079	5.30	20	9	+4.25	−2.74	−3.50
U6	Putative asparate aminotransferase [*H. Vulgare* subsp. *vulgare*]	gi|89511843	45377	5.75	14	4	+1.80	−1.34	+1.36
U7	Predicted pirin-like protein [*Brachypodium distachyon*]	gi|357114735	41337	9.37	17	8	+1.32	−1.72	+1.36
	Energy								
U8	Os06g0133800 [*Oryza sativa japonica*]	gi|115466224	73973	5.44	16	10	+1.07	−1.60	+1.04
U9	ATP synthase beta subunit [*Triticum. monococcum*]	gi|525291	59326	5.56	19	7	+10^6^	0.00	0.00
U10	Aconitate hydratase 3 [*Citrus clementina*]	gi|285309967	98669	5.89	10	9	−1.03	−2.53	+1.04
U11	Fructose-bisphosphate aldolase [*H. vulgare*]	gi|226316443	39071	6.08	29	7	+1.71	+1.10	+1.16
	Cell growth/division								
U12	Predicted proliferating cell nuclear antigen-like [*Brachypodium distachyon*]	gi|357137519	29514	4.61	14	5	+1.69	+1.33	+1.04
	Protein biosynthesis								
U13	Putative elongation factor 1 beta [*H. vulgare*]	gi|7711024	24716	4.52	34	7	+1.86	+1.29	+1.18
	Protein destination/storage								
U14	HSP organizing protein/stress-inducible protein [*Dactylis glomerata*]	gi|281399029	64793	6.11	10	5	+1.07	−1.81	−1.10
U15	Heat shock protein 93-V [*A. Lyrata* subsp. *lyrata*]	gi|297795893	103611	6.36	35	30	+1.06	−1.94	+1.12
U16	Os01g0839700 [*O. sativa japonica*]	gi|115440951	19059	5.34	21	3	−1.03	−1.93	−1.12
U17	Heat shock protein [*Spinacia oleracea*]	gi|425194	71231	5.15	22	12	+2.08	+1.01	+1.10
U18	Cytosolic heat shock protein 90 [*H. vulgare*]	gi|32765549	80654	4.96	27	15	+1.21	−1.99	+1.63
U19	Cytosolic heat shock protein 90 [*H. vulgare*]	gi|32765549	80654	4.96	17	9	+1.01	−1.81	+1.03
	Signal transduction								
U20	RNA-binding Ras-GAP SH3 binding protein [*T. aestivum*]	gi|290579509	45493	4.95	18	7	+1.60	−1.51	−1.11
	Unknown								
U21	Hypothetical protein SORBIDRAFT_10g022570 [*Sorghum bicolor*]	gi|242096224	47231	6.08	13	7	+1.80	−1.57	−1.03
U22	Hypothetical protein LOC100383520 [*Z. mays*]	gi|293336836	77345	6.03	16	11	+1.59	+1.29	+1.26
U23	Predicted protein [*H. vulgare* subsp. *vulgare*]	gi|326513540	58531	6.05	22	40	+2.19	+1.23	−1.72
U24	Predicted protein [*H. vulgare* subsp. *vulgare*]	gi|326513418	35914	5.06	22	9	+1.08	−2.60	−1.36

AASC, Amino acid sequence coverage; MP, Matched peptides.

Protein spot ID refers to numbers in Fig. 7. Accession number of top database match from the NCBInr database. ‘Al *vs* control’ referred to fold variation of Al exposed *vs* unexposed plants. Fold increase and decrease were calculated as Al/control, and –control/Al for up and down -regulated proteins respectively. All ratios shown are statistically significant (*p*<0.05).

**Table 2:** Proteins whose expression were significantly induced (+) in XZ16 roots but down-regulated (−)/unchanged in XZ61, or unchanged in XZ16 but down-regulated in XZ61 under 200 µM Al stress.

Spot ID	Protein name	Accession number	MW, Da	pI	AASC %	MP	Folds (Al *vs* controls)		
							XZ16	XZ61	Dayton
	**Metabolism**								
U25 (U6*)	Putative asparate aminotransferase [*H. vulgare* subsp. *vulgare*]	gi|89511843	45377	5.75	14	4	+1.71	−1.34	+1.20
U26 (U7)	Predicted: pirin-like protein [*Brachypodium distachyon*]	gi|357114735	41337	9.37	17	8	−1.25	−2.05	+1.10
	**Energy**								
U27 (U11)	Fructose-bisphosphate aldolase [*H. vulgare*]	gi|226316443	39071	6.08	29	7	+2.06	−1.22	+1.11
U28	Os09g0535000 [*O. sativa* japonica]	gi|115480367	32715	6.96	34	9	+10^6^	+1.29	0.00
U29	Bp2A protein [*T. turgidum* subsp. *dicoccoides*]	gi|133872436	25759	5.86	27	5	+1.71	−1.72	0.00
U30	Phosphoglycerate mutase [*T. aestivum*]	gi|32400802	29615	5.43	52	14	+1.94	+1.38	+1.74
U31	Enolase (2-phosphoglycerate dehydratase) [*O. sativa* japonica]	gi|780372	48299	5.42	18	7	+1.25	−1.72	+2.53
U32	atp1 [*Secale strictum*]	gi|166165274	53979	6.01	31	14	+1.15	−4.10	+1.29
	**Cell growth/division**								
U33 (U12)	Predicted: proliferating cell nuclear antigen-like [*B. distachyon*]	gi|357137519	29514	4.61	14	5	+2.16	+1.08	−1.15
	**Protein destination/storage**								
U34	Heat shock 70 kda protein [*Z. mays*]	gi|226500540	72989	5.62	10	4	+1.21	−1.97	+1.07
U35 (U17)	Heat shock protein [*Spinacia oleracea*]	gi|425194	71231	5.15	22	12	+1.92	−1.25	−1.01
U36 (U18)	Cytosolic heat shock protein 90 [*H. vulgare*]	gi|32765549	80654	4.96	27	15	+1.12	−2.91	+1.15
U37 (U19)	Cytosolic heat shock protein 90 [*H. vulgare*]	gi|32765549	80654	4.96	17	9	+1.05	−1.76	−1.14
	**Transporters**								
U38	Vacuolar proton-atpase D subunit [*T. aestivum*]	gi|108925894	41321	4.89	46	10	+1.05	−1.66	+1.12
	**Signal transduction**								
U39	14-3-3D protein [*H. vulgare* subsp. *vulgare*]	gi|83271056	28742	4.80	44	9	+1.31	−1.58	+1.10
	**Disease/defense**								
U40	Phenylalanine ammonia-lyase [*Phyllostachys edulis*]	gi|224998176	76278	6.15	19	12	+1.21	−3.50	+1.96
	**Unknown**								
U41	Predicted protein [*H. vulgare* subsp. *vulgare*]	gi|326496891	38099	4.83	25	7	+2.08	−1.79	+1.22
U42	Hypothetical protein Sb01g000380 [*S. bicolor*]	gi|242032147	60974	5.20	21	9	+1.09	−2.03	+1.36
U43 (U23)	Predicted protein [*H. vulgare* subsp. *vulgare*]	gi|326513540	58531	6.05	22	40	+1.84	−1.11	−3.17
U44 (U24)	Predicted protein [*H. vulgare* subsp. *vulgare*]	gi|326513418	35914	5.06	22	9	−1.32	−3.34	−1.27

* , Spot U25 is the same protein spots as U6 identified in 50 µM Al stress.

Protein spot ID refers to numbers in Fig. 7. Accession number of top database match from the NCBInr database. ‘Al *vs* control’ referred to fold variation of Al exposed *vs* unexposed plants. Fold increase and decrease were calculated as Al/control, and –control/Al for up and down -regulated proteins respectively. All ratios shown are statistically significant (*p*<0.05).

Six chaperone-related proteins were identified: heat shock protein (HSP, U17), HSP 93-V (U15), cytosolic HSP 90 (U18 and U19), HSP organizing protein (U14) and HSP 70 kDa (U34). These spots showed normal expression in XZ16 and Dayton, but were down-regulated in XZ61 under Al stress (Tables 1 and 2), indicating that these proteins are involved in Al detoxification in both XZ16 and Dayton.

**Figure 5:**
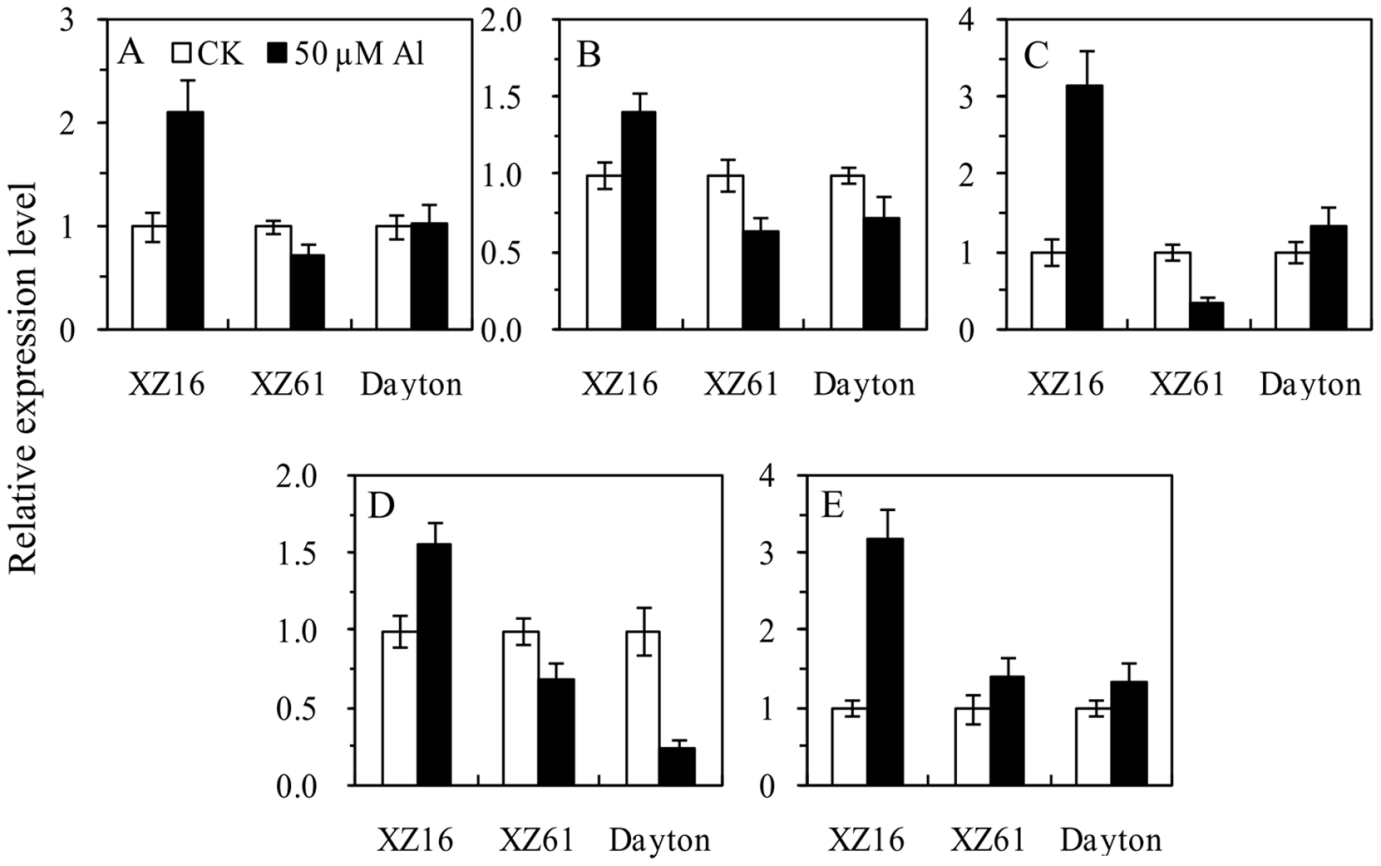
Three root proteins associated with low pH isolated from the root of XZ16. Representative 2-DE maps of root proteins isolated from XZ16 exposed to pH 4.3 for 24 h (A). ‘Spot view’ of the abundance of differentially expressed proteins that were significantly higher expressed (+) in XZ16 compared with Dayton roots under control condition at pH 4.3 (XZ16 *vs* Dayton) but suppressed (−) at pH 6.0 (B).

In addition, expression of three proteins, classified as the energy category (Figure 5 and Table S2) was significantly higher in XZ16 *vs* Dayton under control condition at pH 4.3, and simultaneously suppressed at pH 6.0. They are predicted to be 6-phosphogluconate dehydrogenase decarboxylating-like isoform 1 (6-PGDH) (E1), plastid glutamine synthetase isoform GS2b (E2) and iron deficiency specific clone no. 3 (IDS3) (E3). These proteins are potentially responsible for the low pH tolerance in XZ16 compared with Dayton.

### XZ16 Shows Higher Expression of Genes Corresponding to Al Up-regulated Proteins

**Figure 6:**
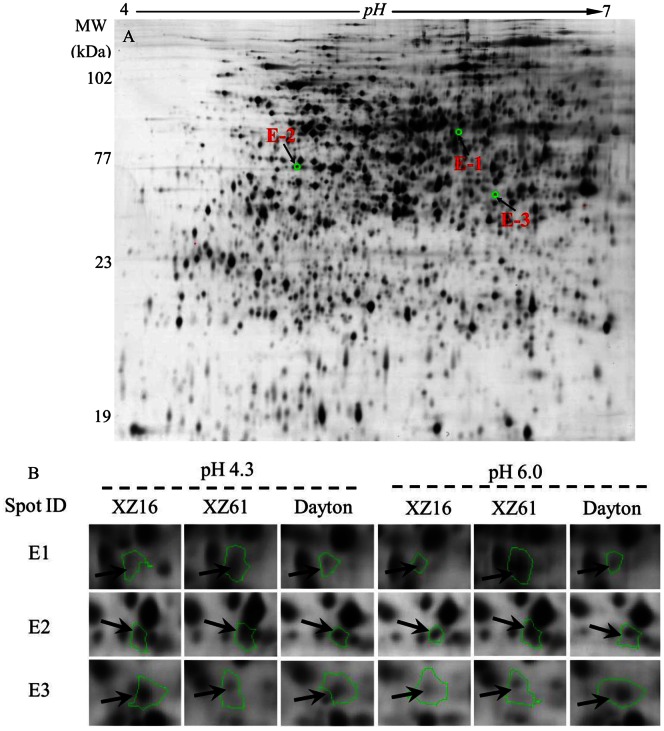
Effect of 50 µM Al on the transcript levels of gene expression encoding selected proteins of three barley genotypes exposed to 50 µM Al for 24 h. (A) S-adenosylmethionine synthase 3 (SAMS3, spot U1); (B) methionine synthase (MeSe, spot U2); (C) glutamine synthetase (GS, spot U4); (D) γ-glutamylcysteine synthetase (γ-GCS, spot U5) and (E) ATP synthase beta subunit proteins in roots (spot U9) of three barley genotypes exposed to 50 µM Al for 24 h. Error bars represent SD values (n = 3).

To determine whether the changes in protein abundance detected by 2-DE were correlated with changes at the transcriptome level, quantitative RT-PCR was performed using RNA isolated from the roots of a separate set of plants treated with 0 or 50 µM Al for 24 h (Figure 6 and Table S2). Transcript levels of five Al-regulated genes including *SAMS3*, *MeSe*, *GS*, *γ-GCS* and *ATP synthase beta subunit* (U1, U2, U4, U5 and U9) were chosen and successfully detected. Among them, *SAM3*, *MeSe*, *GS* and *γ-GCS* were up-regulated in XZ16 and down-regulated in XZ61, following the expression trend detected by 2-DE (Table 1). Fifty micromolar of Al significantly up-regulated *ATP synthase beta subunit* by 3.2 folds in XZ16 compared to the control, but no change was detected in XZ61 and Dayton (Figure 6).

For genome mapping, BLASTn and CAP3 were used to blast the *Brachypodium distachyon* protein databases. The top hits were then mapped to barley chromosome based on barley genome zipper and sequence [18], [19]. Finally, 32 (including 30 of the 35 Al-tolerance related proteins, and 2 of the 3 low pH responsive spots) out of the above 38 proteins were linked to their corresponding candidate genes in barley genome (Table S3). The mapping of these candidate genes provides a short cut for the identification and transformation of Al responsible genes in future.

## Discussion

The current study showed that Al-tolerant Tibetan wild barley XZ16 is characterized by less Al accumulation both in roots and shoots (Figure 1 and Figure S2). Our current data is the first study to identify Al-responsive proteins in Tibetan wild barley (XZ16) using a proteomic approach. Barley is one of the most Al sensitive cereal species and responds to Al toxic ion immediately through releasing organic acid in roots [3], [35]. It is important to successfully induce Al-tolerant-specific-proteins before visible morphological-stress-symptoms for plants to achieve tolerance to Al toxicity. In addition, the micro- and minor- elements, containing in the nutrient solution for the long term Al exposure of 15 days, trends to complicatedly react with Al ion compared to simplified adding Ca^2+^ solution. Furthermore, after long term Al treatment, barley roots (especially in sensitive genotype) is severely damaged, out of vigorousness and partly death, thus not suitable for protein extraction. Therefore, in this study, Al-tolerant-specific-proteins in wild genotype XZ16 were verified under 50 µM or 200 µM Al condition for 24 h. The treatment of 200 µM Al on the plants could provide us the special mechanism (proteins) of Al tolerance in XZ16 in comparison to Dayton or XZ61 under high Al concentration condition. Because Tibetan wild barley XZ16 and XZ61 are the two contrasting genotypes with different Al tolerances, the Al-regulated proteins identified using comparative proteomics will provide a good foundation to elucidate the mechanisms involved in Al- tolerance/resistance in Tibetan wild barley. Here we identified 35 proteins associated with Al-tolerance in wild barley XZ16 (Figures 2, 5 and Tables 1, 2). There were 16 proteins, up-regulated in roots of XZ16 but down-regulated or unaltered in both Dayton and XZ61, indicating their specificity and importance for Al tolerance in XZ16. Among them, four proteins (SAMS3, U1; ATP synthase beta subunit, U9; Os09g0535000, U28; Bp2A protein, U29), were markedly induced by Al stress in XZ16 but not in Dayton, while repressed in XZ61. Obviously, XZ16 has different stress response and defense mechanisms against Al stress as Dayton did. Further examination of these proteins may elucidate the mechanism of Al tolerance in XZ16 and provide new genetic materials for developing Al-tolerant crops. The selected stress-responsive proteins are discussed below according to their function.

### Metabolism Category

**Figure 7:**
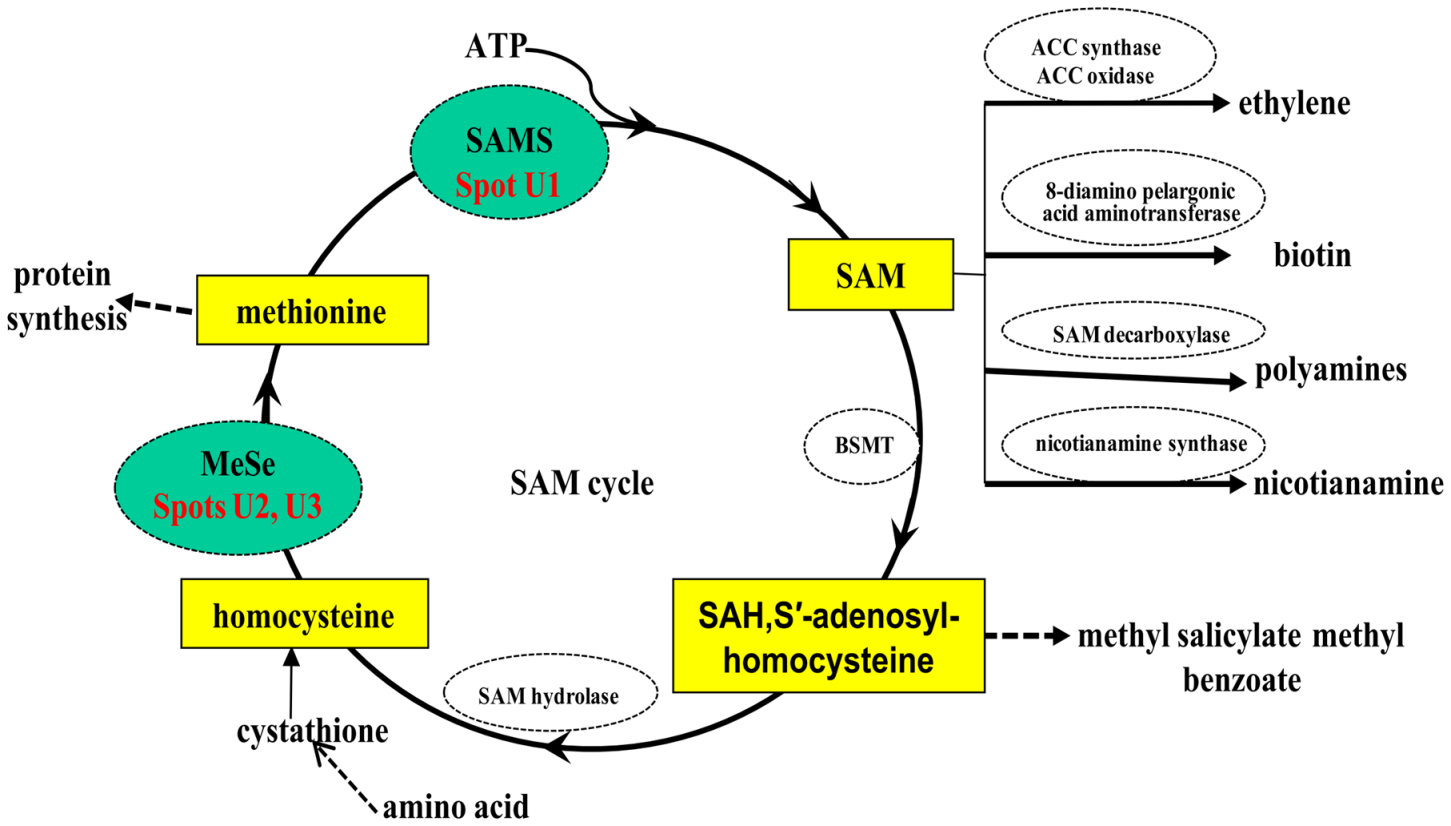
Two of indentified SAM cycle-related enzyme proteins SAMS and MeSe (green oval-shaped box) associated with Al-tolerance in Tibetan wild barley XZ16. SAM regeneration and utilization reactions of the SAM cycle are depicted. Enzymes of SAM cycle are: MeSe, methionine synthase; SAMS, SAM synthetase; BSMT, benzoic acid/salicylic acid: S-adenosyl-methionine carboxyl methyltransferase; SAM hydrolase. SAH, S′-adenosyl-homocysteine; ACC, 1-aminocyclopropane-1-carboxylate. ACCS, 1-aminocyclopropane-1-carboxylic acid synthase.

In the root proteomic analyses, seven of the identified proteins are involved in metabolism: SAMS3 (U1), methionine synthase (MeSe, U2 and U3), glutamine synthetase (GS, U4), γ-glutamylcysteine synthetase (γ-GCS, U5), putative aspartate aminotransferase (AST, U6) and predicted pirin-like protein (U7). SAMS, as an essential enzyme in cellular metabolism, has been long regarded as a ‘housekeeping’ function and it catalyzes the nucleophilic substitution reaction between methionine and ATP into SAM (S′-adenosyl-L-methionine) (Figure 7). SAM serves as an aminopropyl and methyl donor for ethylene and polyamine (PAs) [20], [21], [22]. It is well documented that ethylene and PAs are involved in response to biotic and abiotic stresses in plants [23], [24]. Thus SAMS plays an important role in Spd and Spm biosynthesis and stress response in plants [25], [26]. Qi *et al.* [27] found that over-expression of *Suadea salsa SAMS2* in transgenic tobacco plants leads to an increase in polyamine (PAs) content and enhancement of salt tolerance. In the present study, SAMS3 was up-regulated in XZ16 but not expressed in Dayton and repressed in XZ61 under 50 µM Al stress, highlighting the function of this protein in Al tolerance of XZ16. Although SAM is the precursor of ethylene, the key enzyme of ethylene synthesis is ACCS [28] (Figure 7). Since ACCS was not identified in this study, further research is required to determine whether up-regulation of SAMS is involved in ethylene-mediated inhibition of root growth and/or the alteration of cell wall and polymer structures in roots under Al stress. On the other hand, *SAMS3* was expressed normally in Dayton under Al stress as determined by qRT-PCR (Figure 6). Therefore, Al-induced expression of *SAMS3* is likely to be regulated at the post-transcriptional or translational level in Dayton.

In addition, we also observed that MeSe was depressed in XZ61 (U2 and U3), but not altered in XZ16 under Al stress. Higher MeSe levels would increase the amount of methionine for SAM synthesis (Figure 7). Enhanced accumulation of MeSe contributes to increase PAs biosynthesis, which is critical to plant survival in many environmental stresses [29]. The decreased accumulation of MeSe in XZ61 indicates that Al stress triggered damages in barley roots may be associated with the modification of amino acid metabolism and synthesis of other amino acids derived metabolites. The next important questions is how SAMS3 and MeSe elaborate to cope with Al toxicity and which metabolism factors, e.g., SAM, ethylene, PAs (spermidine or spermine), are responsible for Al detoxification in conjunction with SAMS3 and MeSe. Therefore, it is necessary to investigate the accumulation of related metabolite and enzymatic activities of the SAMS family for a better understanding of their functions in the Al stress response.

Two glutathione (GSH) synthesis proteins γ-GCS and GS (U4 and U5), play a pivotal role in various metabolic processes involved in plant growth and development and stress responses including Al toxicity [30], [31]. The pirin-like protein (U7 and U26) plays important roles in a number of different biological processes, however, its physiological role in plants remains unclear [32]. Aspartate aminotransferase (AST, U6 and U25) is a key antioxidative enzyme for detoxifying reactive oxygen species (ROS) under abiotic stresses [33]. Similarly, up-regulation of AST expression was reported in Arabidopsis in response to Al stress [34]. Under Al stress, γ-GCS (U5), GS and AST were significantly increased in XZ16, whereas dramatically decreased in XZ61 and Dayton (Table 1), again indicating that there a unique mechanism in Al tolerance for the Tibetan wild barley genotype.

### Energy Category

Similar to the proteomic results of Zhou *et al.* [10], ATP synthase beta subunit (U9) was only detected in XZ16 after 24 h of 50 µM Al stress (Table 1), indicating that the energy provided to ATPase for active Al efflux and detoxification in XZ16 was increased when exposed to Al stress. qRT-PCR of *ATP synthase beta subunit* confirmed a remarkable increase in XZ16 but not in X61 and Dayton under 50 µM Al (Figure 6 **)**. Therefore, we conclude that ATP synthase beta subunit significantly contributes to the ATPase-mediated active Al efflux and detoxification, and is regulated at both transcriptional and translational level in XZ16 in response to Al.

In addition to ATP synthase beta subunit, we also identified another type of ATPase, atp1 (U32), which was markedly down-regulated in XZ61 after Al stress, but remained unchanged in XZ16 and Dayton (Table 2). The efflux of Al-induced citrate and malate is usually mediated by anion channels and ATPase-driven active co-transporters [35], [36]. Interestingly, a significant increase in ATP synthase beta subunit and unchanged levels of atp1 in XZ16 were consistent with higher root citrate and malate efflux, whereas the lack of ATP synthase beta subunit expression and the Al-induced severe inhibition of atp1 in XZ61 were associated with lower citrate and malate secretion. These results firmly suggest that high level of ATP synthase beta subunit and atp1 in XZ16, unlike that in Dayton, may partly contribute to the active OA transport and secretion during protection from Al-toxicity. Thus, we may speculate that a novel protein synthesis of ATP synthase beta subunit may be involved only in Al inducing organic acid secretion in Tibetan wild barley XZ16. Obviously, it deserves a more detailed investigation in the future.

A BLAST search revealed that Os09g0535000 (U28) is a homologue of triosephosphate isomerase (TPI). TPI was reported to be induced in rice [37] and maize [38] under drought stress, indicating the importance of cellular homeostasis maintenance and emphasizing the role of this protein in energy production. Enolase (ENO, U31) is responsive to salt, low and high temperature and anaerobic stresses [8], [39]. Spot U10, identified as aconitate hydratase 3 (Aco3), plays a role in regulating resistance to oxidative stress and cell death in *Arabidopsis* and *Nicotiana benthamiana* [40]. Spot U29, the Bp2A protein, was first identified in the wheat genome [41]. The present study is the first to examine Bp2A (U29) expression under Al stress. However, the function of this protein and its direct involvement in Al tolerance are poorly understood. Therefore, the mechanisms underlying the differential expression of this protein in different barley genotypes should be further explored.

In the glycolytic pathway, the levels of other Al stress responsive proteins fructose-bisphosphate aldolase (FBA, U11 and U27) and phosphoglycerate mutase (PGM, U30) were increased in XZ16 under Al treatments, consistent with the findings of Fukuda *et al.* [42] in rice under Al stress and Yan *et al.* [43] in the rice response to chilling. Down-regulation of Os06g0133800 (U8), a homologue to transketolase (TK), in XZ61 may reversibly inhibit ribose 5-phosphate, which is a substrate for nucleic acid synthesis associated with the synthesis of RNA under Al stress [42].

**Figure 8:**
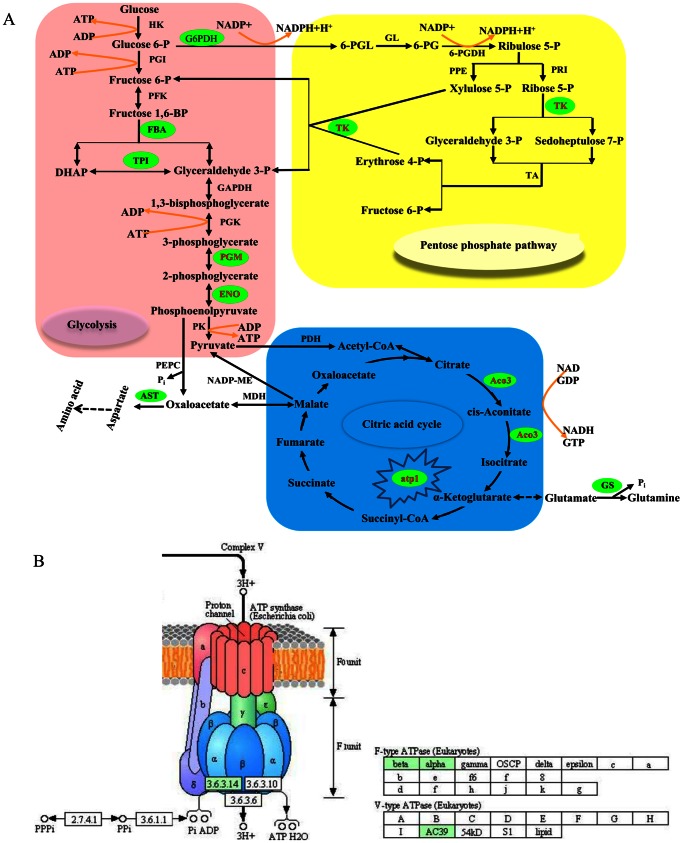
Some of indentified proteins (green oval-shaped box, cycle-related enzymes) associated with Al-tolerance involving in the metabolic pathways of glycolysis (purple box), pentose phosphate pathway (yellow box), and citric acid cycle (blue box) in Tibetan wild barley XZ16 (A). Searching via the KEGG (Kyoto Encyclopedia of Genes and Genomes), ATP synthase beta subunit (spot U9), atp1 (F-tyep ATP synthase α subunit, spot U32) and V-ATPase (V-type ATPase AC39, spot 38) were represented by “3.6.3.14” in oxidative phosphorylation (B). Glucose 6-P either proceeds through glycolysis (participated by FBA, TPI, PGM and ENO) or the PPP (participated by TK) to produce pyruvate, which is converted to acetyl-CoA, entering the TCA cycle and producing citrate and malate (participated by Aco3). atp1 belongs to mitochondrial membrane ATP synthase generates ATP from ADP in the presence of a proton gradient across the membrane. Glutamine and other amino acids also feed by the TCA cycle; for example, α-ketoglutarate can converte glutamate, which is converted to glutamine by GS; malate can be converted oxaloacetate, which is coverted to aspartate through AST. Abbreviation: HK, hexokinase; PGI, phosphateisomerase; PFK, phosphofructokinase; BP, bisphoshate; FBA, fructose-bisphosphate aldolase; TPI, triose phosphate isomerase; GAPDH, glyceraldehyde-3-phosphate dehydrogenase; PGK, phosphoglycerate kinase; PGM, phosphoglycerate mutase; ENO, enolase; PK, pyruvate kinase; G6PDH, glyceraladehyde 6-phosphate dehydrogenase; 6-PDL, 6-phosphogluconolactone; GL, gluconolactonase; 6-PG, 6-phosphogluconate; 6-PGDH, 6-phosphogluconate dehydrogenase; PPE, pentose phosphate epimerase; PRI, phosphoriboisomerase; TK, transketolase; TA, transaldolase; PDH, pyruvate dehydrogenase; CoA, coenzyme A; Aco3, aconitate hydratase 3; GS, glutamine synthetase; PEPC, phosphoenolpyruvate carboxylase; MDH, malate dehydrogenase; NADP-ME, NADP-malic enzyme; AST, asparate aminotransferase.

Taken together, up-regulation of FBA, PGM, TPI, Bp2A, and ATP synthase beta subunit (U11, U27, U30, U28, U29 and U9) and maintaining the normal expression levels of the other four energy proteins in XZ16 might help to produce more energy needed in the defense processes under Al stress conditions. The higher abundance of all these enzymes catalyzing various reactions in glycolysis, pentose phosphate pathway, and citric acid cycle in roots of Al resistant genotypes, suggest less Al-triggered disruption of energy metabolism in XZ16 (Figure 8). Up-regulation of ATP synthase beta subunit, Bp2A, TPI and FBA is of particular importance to XZ16 relative to Dayton.

### Cell Growth/Division and Protein Biosyntheses Category

Proliferating cell nuclear antigen (PCNA) is an essential component in eukaryotic DNA synthesis [44]. PCNA interacts with many proteins and participates in a variety of metabolic processes, such as cell cycle control, nucleotide excision repair and post-replication mismatch repair [45]. Translation elongation factor 1β (EF1B, U13) is a highly conserved protein that catalyzes the exchange of bound GDP for GTP on EF-1α, a required step to ensure continued protein synthesis. In our study, PCNA (U12 and U33) and EF1B (U13) were up-regulated in in roots of XZ16, but were unchanged in XZ61 and Dayton. It suggests that XZ16 might possess a high actitity of DNA replication machinery in response to Al stress, and EF1B may play an important role in maintaining the root elongation rate of XZ16 under Al stress.

In addition, the other specific protein up-regulated in XZ16 is the RNA-binding Ras-GAP SH3 binding protein (G3BP, signal transduction category, U20). Zhou *et al.* [46] reported that the expression of the *G3BP* gene was associated with fertility conversion in male-sterile wheat. However, the exact function of G3BP in RasGAP-dependent signaling remains to be defined. These results provide a starting point for further investigation into the functions of these proteins using genetic and other approaches.

In conclusion, the response and defense mechanisms of Al stress in XZ16 appear different from those of Dayton, as reflected by the different expressions of these specific proteins associated with Al tolerance under Al stress between XZ16 and Dayton or XZ61. There are four proteins (i.e. SAMS3, ATP synthase beta subunit, TPI, Bp2A protein), which are exclusively expressed in XZ16 not in Dayton and XZ61 under Al stress, indicating their crucial role in development of Al stress tolerance in XZ16, and novelty of genetic resource for Al-tolerance. In addition, as the functions of some differentially expressed proteins and their direct involvement in stress tolerance are poorly understood, further studies are warranted to elucidate the underlying molecular and metabolic pathways for better understanding the mechanisms involved in Al-tolerance of wild barley and providing new genetic resources in Al-tolerant crop breeding.

## Supporting Information

Figure S1 **Effect of Al stress on root (A) and individual plant dry weight (B) of barely seedlings of XZ16, XZ61 and Dayton.**
(DOC)

Figure S2
**Al accumulation in roots (A) and shoots (B) and transferring rate from root to shoot (C) of barley seedlings of XZ16 (□), XZ61 (▪) and Dayton (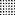).**
(DOC)

Table S1 **Oligonucleotides used as primers for real-time RT-PCR.**
(DOC)

Table S2 **Proteins whose expression were significantly higher expressed (+) in XZ16 compared with Dayton roots under control condition at pH 4.3 (XZ16** ***vs*** **Dayton) but suppressed (−) at pH 6.0.**
(DOC)

Table S3 **Thirty-two proteins (including 2 low pH responsive proteins E1 and E2) corresponding candidates were mapped to chromosome based on barley genome zipper information.**
(DOC)
