# Supplementary material for: Comparative Proteomic Analysis of Aluminum Tolerance in Tibetan Wild and Cultivated Barleys
Source: PLoS One. 2013 May 14;8(5):e63428. doi: 10.1371/journal.pone.0063428 (PMC3653947; doi:10.1371/journal.pone.0063428)
Supplement: Figure S1 — Effect of Al stress on root (A) and individual plant dry weight (B) of barely seedlings of XZ16, XZ61 and Dayton. (DOC) [file pone.0063428.s001.doc]

Root DW per plant (mg)

A

*

*

*

Individual plant DW (mg)

B

*

**Figure S1. Effect of Al stress on root (A) and individual plant dry weight (B) of barely seedlings of XZ16, XZ61 and Dayton.** Plants were harvested after exposure to 50 and 200 µM Al (pH 4.3) for 24 h, oven-dried for 2 days at 70°C and weighed. Data are means ± SD (n=8). *, Signiﬁcant difference, p < 0.05.
