# Supplementary material for: Comparative Proteomic Analysis of Aluminum Tolerance in Tibetan Wild and Cultivated Barleys
Source: PLoS One. 2013 May 14;8(5):e63428. doi: 10.1371/journal.pone.0063428 (PMC3653947; doi:10.1371/journal.pone.0063428)
Supplement: Figure S2 — Al accumulation in roots (A) and shoots (B) and transferring rate from root to shoot (C) of barley seedlings of XZ16 (□), XZ61 (▪) and Dayton (). (DOC) [file pone.0063428.s002.doc]

Transferring rate (%)

B

A

Al accumulation (µg plant^-1^)

C

Solution Al level (μM)

**Figure S2. Al accumulation in roots (A) and shoots (B) and transferring rate from root to shoot (C) of barley seedlings of XZ16 (□), XZ61 (■) and Dayton (░).** Seedlings were subjected to 0, 50 and 200 µM AlCl3 in 0.5 mM CaCl2 solution at pH 4.3 for 24 h, respectively, and then roots and shoots were collected for Al determination. Data are means ± SD (n=5).
