# Supplementary material for: Comparative Proteomic Analysis of Aluminum Tolerance in Tibetan Wild and Cultivated Barleys
Source: PLoS One. 2013 May 14;8(5):e63428. doi: 10.1371/journal.pone.0063428 (PMC3653947; doi:10.1371/journal.pone.0063428)
Supplement: Table S1 — Oligonucleotides used as primers for real-time RT-PCR. (DOC) [file pone.0063428.s003.doc]

**Table S1. Oligonucleotides used as primers for real-time RT-PCR**.

| **Gene** | **Primer orientation** | **Primer sequence (**5'-3'**)** | **Amplicon size** |
| --- | --- | --- | --- |
| *SAM3* | Forward | CAACATGGTCATGGTCTTCG | 165 |
|  | Reverse | ATGTCAGGGGATTGTTGCTC |  |
| *MeSe* | Forward | CGTCCCAGACCGTTACTCAT | 234 |
|  | Reverse | AGCACTGGGACGGTATCAAC |  |
| *GS* | Forward | TTTCTGCTGGTGACCAAGTG | 221 |
|  | Reverse | GATGTGCTCCTTGTGCTTCA |  |
| *γ-GCS* | Forward | AAACATTGCGCCCTATAACG | 248 |
|  | Reverse | TCCCAATTCCTCTCCAACTG |  |
| *ATP synthase beta subunit* | Forward | TTTCTCTGTGTTCGCTGGTG | 224 |
