# Supplementary material for: Comparative Proteomic Analysis of Aluminum Tolerance in Tibetan Wild and Cultivated Barleys
Source: PLoS One. 2013 May 14;8(5):e63428. doi: 10.1371/journal.pone.0063428 (PMC3653947; doi:10.1371/journal.pone.0063428)
Supplement: Table S2 — Proteins whose expression were significantly higher expressed (+) in XZ16 compared with Dayton roots under control condition at pH 4.3 (XZ16 vs Dayton) but suppressed (−) at pH 6.0. (DOC) [file pone.0063428.s004.doc]

**Table S2. Proteins whose expression were significantly higher expressed (+) in XZ16 compared with Dayton roots under control condition at pH 4.3 (XZ16 *vs* Dayton) but suppressed** (-) at pH 6.0.

| Spot ID | Protein name | Accession number | MW Da | pI | AASC  % | MP | Folds increase (+) or decrease (-) | | | | |
| --- | --- | --- | --- | --- | --- | --- | --- | --- | --- | --- | --- |
| XZ16 *vs* Dayton | |  | XZ16 *vs* XZ61 | |
| 4.3* | 6.0 | 4.3 | | 6.0 |
| E1 | Predicted: 6-phosphogluconate dehydrogenase, decarboxylating-like isoform 1 [*Brachypodium distachyon*] | [gi|357110692](http://www.matrixscience.com/cgi/master_results.pl?file=..%2Fdata%2F20120105%2FFtoTfzTOL.dat&REPTYPE=protein&_sigthreshold=0.05&REPORT=10#Hit1) | 52865 | 5.61 | 23 | 13 | +1.50 | -1.75 | -2.62 | | -7.13 |
| E2 | plastid glutamine synthetase isoform GS2b [*T. aestivum*] | [gi|71362638](http://www.matrixscience.com/cgi/master_results.pl?file=..%2Fdata%2F20120105%2FFtoTfzeET.dat&REPTYPE=protein&_sigthreshold=0.05&REPORT=10#Hit1) | 46964 | 6.04 | 23 | 7 | +1.82 | -1.90 | -1.20 | | -2.41 |
| E3 | IDS3 (iron deficiency specific clone no. 3) [*H. vulgare subsp. vulgare*] | [gi|9711238](http://202.116.111.30/mascot/cgi/master_results.pl?file=..%2Fdata%2F20120130%2FF039178.dat&REPTYPE=protein&_sigthreshold=0.05&REPORT=10&_server_mudpit_switch=99999999&_ignoreionsscorebelow=0&_showsubsets=0&_showpopups=TRUE&_sortunassigned=scoredown&_requireboldred=0#Hit1) | 38346 | 5.81 | 33 | 11 | +1.94 | -1.86 | +1.81 | | +1.33 |

AASC, Amino acid sequence coverage; MP, Matched peptides. *, solution pH.

Protein spot ID refers to numbers in Fig 6. Accession number of top database match from the NCBInr database. Fold increase and decrease were calculated as pH 4.3 / pH 6.0, and –pH 4.3 / pH 6.0 for up and down-regulated proteins, respectively.
