# Supplementary material for: Comparative Proteomic Analysis of Aluminum Tolerance in Tibetan Wild and Cultivated Barleys
Source: PLoS One. 2013 May 14;8(5):e63428. doi: 10.1371/journal.pone.0063428 (PMC3653947; doi:10.1371/journal.pone.0063428)
Supplement: Table S3 — Thirty-two proteins (including 2 low pH responsive proteins E1 and E2) corresponding candidates were mapped to chromosome based on barley genome zipper information. (DOC) [file pone.0063428.s005.doc]

**Table S3. Thirty-two proteins (including 2 low pH responsive proteins E1 and E2) corresponding candidates were mapped to chromosome based on barley genome zipper information.**

| Chromosome | Marker | Cm | Protein name | Spots ID | Brachypodium SeqID |
| --- | --- | --- | --- | --- | --- |
| 1H | 1_0314 | 45.13 | γ-glutamylcysteine synthetase | U5 | Bradi2g38340.1 |
|  | 1-1409-1-0729 | 115.9-116.3 | ATP synthase beta subunit | U9 | Bradi2g17290.1 |
|  | 2_1192-1_0471 | 88.23-88.23 | Os01g0839700 | U16 | Bradi2g23240.1 |
|  | 2_0594-2_0138 | 136.31-137.83 | Predicted proliferating cell nuclear antigen-like | U33 (U12) | Bradi2g14500.1 |
| 2H | 2_0387-2_0602 | 54.95-54.95 | Putative elongation factor 1 beta | U13 | Bradi1g21550.2 |
|  | 2_0715-3_0248 | 133.94-136.8 | atp1 | U32 | Bradi5g23842.1 |
|  | 1_0436-2_0690 | 62.82-62.82 | 14-3-3D protein | U39 | Bradi5g12510.2 |
|  | 2_0261 | 10.06 | Hypothetical protein Sb01g000380 | U42 | Bradi5g02890.3 |
| 3H | 1_0680 | 117.87 | Fructose-bisphosphate aldolase | U27 | Bradi2g58050.1 |
|  | 3_0663-3_0325 | 89.31-90.48 | Bp2A protein,  phosphoglycerate mutase | U29,  U30 | Bradi2g53340.3 |
|  | 3_0909 | 98.49 | Vacuolar proton-ATPase D subunit | U38 | Bradi2g56260.1 |
|  | 1_0918 | 123.68 | predicted protein | U44 | Bradi2g57500.1 |
| 4H | 3_0906 | 66 | plastid glutamine synthetase isoform GS2b | E2 | Bradi1g69530.1 |
|  | 3_0907 | 28.4 | Glutamine synthetase | U4 | Bradi1g11450.1 |
|  | 1_0271 | 96.59 | Aconitase-iron regulated protein 1 | U10 | Bradi1g75960.1 |
|  | 1_0606-2_0072 | 67.46-67.46 | Predicted protein | U43 | Bradi1g70180.1 |
| 5H | 2_1426-3_0531 | 27-27.72 | Methionine synthase | U2, U3 | Bradi4g01200.2 |
|  | 2_0736 | 85.93 | Putative asparate minotransferase | U25 (U6) | Bradi4g31960.1 |
|  | 2_1503 | 183.6 | Predicted pirin-like protein-like protein | U26 (U7) | Bradi1g01870.1 |
|  | 3_0456-1_1341 | 113.11-113.83 | Os09g0535000 | U28 | Bradi4g36310.1 |
|  | 2_0708-1_0481 | 51.3-51.3 | Predicted protein | U41 | Bradi4g40860.1 |
| 6H | 1_0270 | 60.23 | hsp organizing protein/stress-inducible protein | U14 | Bradi3g50110.1 |
|  | 2_0654-1_0815 | 84.51-85.16 | Heat shock 70 kda protein,  heat shock protein | U34,  U35 | Bradi3g57450.1 |
|  | 2_0184-1_1143 | 56.48-56.48 | Phenylanlanine ammonia-lyase | U40 | Bradi3g49260.1 |
| 7H | 3_0600 | 78.22 | Predicted 6-phosphogluconate dehydrogenase, decarboxylating-like isoform 1 | E1 | Bradi3g17120.1 |
|  | 1_0025 | 21.13 | Os06g0133800 | U8 | Bradi1g51670.1 |
|  | 1_0089 | 87.97 | Hypothetical protein SORBIDRAFT_10g022570 | U21 | Bradi1g37490.1 |
|  | 1_0698 | 78.22 | Hypothetical protein LOC100383520 | U22 | Bradi3g41550.1 |
|  | 1_0025-2_0495 | 21.13-25.7 | Enolase (2-phosphoglycerate dehydratase) | U31 | Bradi1g51540.1 |
|  | 2_0827-3_1120 | 73.75-74.52 | Cytosolic heat shock protein 90 | U18, U19, U36, U37 | Bradi3g39590.1 |
